# Root cause analysis of accidents and examining their interrelations from the perspective of workers, supervisors, and safety officers

**DOI:** 10.1371/journal.pone.0334968

**Published:** 2025-11-05

**Authors:** Neda Molamehdizadeh, Gholam Hossein Halvani, Hossein Ebrahimi, Ali Asghar Farshad, Seyedeh Melika Kharghani Moghadam, Tahereh Eskandari

**Affiliations:** 1 Department of Occupational Health and Safety Engineering, School of Public Health, Iran University of Medical Sciences, Tehran, Iran; 2 Department of Occupational Health and safety Engineering, School of Public Health, Shahid Sadoughi University of Medical Sciences, Yaz, Iran; 3 Occupational Health Research Center, Department of Occupational Health and Safety Engineering, School of Public Health, Iran University of Medical Sciences, Tehran, Iran; 4 Department of Health Services, School of Public Health, Iran University of Medical Sciences, Tehran, Iran; Arak University of Medical Sciences, IRAN, ISLAMIC REPUBLIC OF

## Abstract

Identifying the root causes of accidents and analyzing their causal relationships is a fundamental step in designing effective preventive strategies. This study aimed to uncover the root causes of mining accidents and determine the interactions among them from the perspectives of workers, supervisors, and safety officers using a mixed qualitative-quantitative approach and the fuzzy DEMATEL method. The study was designed as a mixed-method approach (qualitative-quantitative). In the qualitative phase, a total of 69 interviews were conducted (23 with workers, 21 with supervisors, and 25 with safety officers) to identify and categorize the root causes of accidents. In the quantitative phase, 33 participants (11 from each group) took part in expert panels where the fuzzy DEMATEL method was employed to analyze the relationships among the factors. The qualitative phase results revealed that workers primarily pointed to operational deficiencies, equipment issues, and workplace conditions. Supervisors emphasized human behavior, psychological stress, and a lack of safety culture, while safety officers highlighted managerial weaknesses and inefficient communication structures. The quantitative phase results identified management as the primary and most influential factor, whereas other factors, including humans, machinery, environment, and materials, predominantly appeared as dependent factors. This study’s findings suggest that understanding and analyzing the causal relationships among factors, coupled with integrating diverse perspectives, can aid in designing effective preventive strategies and reducing mining accidents. This approach enhances safety, productivity, and job satisfaction.

## 1- Introduction

In every occupational field, due to environmental conditions and materials there are risks that threats the health of employees and workers [1]. Mines face significantly greater hazards than most other industries because of the nature of their operations [1]. The OSHA organization reported that between 1995 and 2006, 113 fatal mining accidents occurred. Additionally, studies have shown that fatal injury rates for miners are 7 to –10 times higher than the average for the workforce in the United States, Australia, and New Zealand [2].

Mining accidents not only result in extensive loss of life and financial damage but also negatively impact productivity, employee job satisfaction, and the public image of the mining industry [3,4]. Research has highlighted that accurately identifying the root causes of accidents is a crucial step in preventing similar incidents in the future [1,5].

Root causes differ from surface-level factors, which are often recognized as symptoms of accidents. They represent deeper underlying elements that directly contribute to hazardous conditions [6]. These factors are generally categorized into management, human, equipment and machinery, environmental conditions, and materials [2,7]. For example, managerial decisions regarding policymaking, preventive maintenance, or resource allocation have a direct impact on workplace safety [2]. When these issues are neglected, accidents may occur that appear to result from human error or equipment failure but are actually rooted in systemic managerial inefficiencies [1,3]. Prior studies have shown that identifying root causes is essential for developing effective and sustainable preventive measures [1,4].

The perspectives of different occupational groups regarding the root causes of mining accidents play a pivotal role in accurately analyzing these incidents and designing preventive strategies. The importance of diverse viewpoints lies in the fact that each group focuses on specific aspects of the safety system that may be overlooked by others. For instance, workers are often adept at identifying technical deficiencies, whereas safety officers tend to emphasize managerial policies. Integrating these perspectives enables a more comprehensive identification of root cause [4,6]. Determining the relationship between these factors helps to identify the complex interactions between them and formulate more effective strategies for prevention [8].

Most accidents result from the interaction of multiple factors rather than a single cause. For example, machinery failure may stem from poor managerial policies regarding maintenance or human error in equipment operation. Identifying these causal relationships allows organizations to address multiple interactions rather than focusing on a single factor [9].

Determining the relationships between root causes facilitates a systematic analysis of accidents. This approach emphasizes identifying systemic factors that may lead to incidents over time. For instance, a poor safety culture can influence both human behavior and managerial decision-making [10].

Examining causal relationships helps identify the most influential factors, allowing preventive actions to focus on them. Factors identified as root causes require greater emphasis in safety management, as their impact on other factors is also significant [11].

Causal relationship analysis using the fuzzy DEMATEL method is a modern and effective approach for identifying and modeling complex interactions among various factors in multidimensional systems. This method combines the network analysis approach of DEMATEL with fuzzy logic and is specifically designed for situations where relationships among variables are nonlinear, ambiguous, or multi-criteria in nature [12,13].

One of the key advantages of the fuzzy DEMATEL method is its ability to identify critical factors within a system. These factors are divided into two categories: influencing factors, recognized as causes, and influenced factors, considered effects. This distinction helps decision-makers focus on altering or improving causal factors, which can have a widespread impact on the system [14,15].

Identifying the root causes of accidents from diverse perspectives and determining the causal relationships among these causes can play a significant role in accident management. Accordingly, this study aimed to uncover root causes and examine their interrelations from the viewpoints of workers, supervisors, and safety officers using a mixed-method qualitative-fuzzy DEMATEL approach in a surface mine.

## 2- Materials and methods

This study was conducted in two phases. In the first phase, the root causes of mining accidents were identified through interviews with workers, supervisors, and safety officers. In the second phase, the interrelations and interactions among the root causes were analyzed and quantified using the fuzzy DEMATEL method.

### 2-1- Phase 1: Qualitative study

In this phase, the root causes of mining accidents were examined from the perspectives of workers, supervisors, and safety experts. This stage was planned and implemented using an exploratory-sequential approach. The process of this phase is detailed as follows:

#### 2-1-1- Sampling.

A purposive sampling method was employed to select participants, aiming to thoroughly explore aspects related to the study population. The number of participants for each group was set between 10 and 20 [14]. Interviews continued until data saturation was reached. Permission was obtained from the mine management to identify eligible participants. Individuals meeting the selection criteria (minimum of 5 years of work experience, no conflicts of interest, and willingness to participate) were included. Study objectives were explained during face-to-face sessions, and upon their consent, participants were enrolled in the study.

#### 2-1-2- Conducting interviews.

To conduct the study, ethical approval was first obtained from the Research Deputy of Iran University of Medical Sciences (IR.IUMS.REC.1401.923). After selecting the participants, the purpose of the study was explained to them by the researcher, and verbal consent to participate in the study was obtained. If any individuals declined to participate, other eligible participants were selected as replacements. After obtaining verbal consent from the participants, the Interviews were conducted from 5/07/2024 to 1/08/2024.

The interviews were conducted in a semi-structured format. A single open-ended guiding question was used: “From your perspective, what are the root causes of accidents in the mine?” The interviewer provided prompts or clarifications when necessary to encourage participants to elaborate on their experiences and views.

Interviews were conducted in a quiet and disturbance-free environment to ensure participants could freely express their opinions. All interviews were conducted individually by one of the researchers and lasted between 60 and 90 minutes. Participants’ responses were recorded using a voice recorder (Tesco Model 908TR) alongside handwritten notes. At the end of each interview, the researcher summarized the responses and asked participants to clarify any potential inconsistencies. Initial data analysis was performed continuously until data saturation was achieved.

#### 2-1-3- Data analysis.

Interview data were analyzed using a qualitative content analysis approach. After transcribing the interviews, a three-member panel of safety and qualitative research experts was convened to ensure rigorous data analysis. Coding was used to extract and categorize factors from the interviews. Two types of coding—open coding and axial coding—were employed [15,16]. Open coding involved naming factors without restrictions, while axial coding linked variables to main and subcategories [15,17].

To confirm the main themes, independent analyses were conducted by two other researchers, and consensus was reached. The analysis results were shared with participants (member checking) to incorporate their feedback into the findings. A total of 23 interviews with workers, 21 with supervisors, and 25 with safety experts were conducted.

#### 2-1-4- Validity and reliability of qualitative data.

Four criteria—credibility, dependability, confirmability, and transferability—were used to evaluate the validity, precision, and reliability of the qualitative data:

**1- Credibility**: Ensuring the extracted data accurately reflected participants’ statements without subjective interpretation. This was achieved through strategies such as member checking.

**2- Dependability**: Referring to the consistency or repeatability of findings under similar conditions. The researcher ensured consistency by obtaining feedback from participants and conducting team reviews.

**3- Confirmability**: Demonstrating that findings are rooted in the data and derived logically from the sources. This was ensured through audits by external reviewers and participant feedback.

**4- Transferability**: Relating to the theoretical parameters of the research and the applicability of findings in different contexts. To enhance transferability, diverse participant experiences across age, education, cultural, and social backgrounds were considered. This diversity improved the potential applicability of findings to other settings. Additionally, reports were reviewed and approved by colleagues to ensure transferability.

### 2-2- Phase 2: Quantitative study

After identifying the root causes of accidents from the perspectives of workers, supervisors, and safety experts, the next phase of the study focused on quantifying and establishing relationships between these factors. The fuzzy DEMATEL technique was employed to analyze the relationships among factors. Given the differing perspectives of workers, supervisors, and safety officers regarding the root causes of accidents, the factors were categorized into five groups: management, human, equipment and machinery, workplace environment, and materials. These categories were analyzed from the viewpoints of the three study groups.

DEMATEL is recognized as an efficient and reliable tool for analyzing relationships among factors in complex systems. By incorporating expert opinions, this method identifies and evaluates interactions among factors, making it highly effective for multi-criteria decision-making [18]. Furthermore, the fuzzy version of this method adds value by addressing uncertainties in human judgment, thereby enhancing the precision and flexibility of analyses [19]. In this study, the fuzzy DEMATEL method pursued three main objectives includes: 1-Calculating the correlation matrix among factors, 2- Identifying causal factors and determining the influence level of each cause and 3- Developing a causal model of the factors influencing mining accidents. This phase consisted of the following steps.

#### 2-2-1- Formation of expert panels.

There is no definitive guideline for determining the size or composition of expert panels across studies. However, the panel should consist of selected experts chosen based on their relevant knowledge and experience, with no strict limitation on the number of members. According to Hogarth, the optimal panel size for multi-criteria decision-making is between six and twelve members [20]. In most cases, a typical panel consists of 10 to 30 experts [21].

Given the study’s initial goal of exploring the root causes of accidents from the perspectives of workers, supervisors, and safety officers, three independent panels were formed for these groups. Panel members were selected from those interviewed in the previous phase, ensuring familiarity and expertise on the study topic. To form the panels, all previously interviewed participants were contacted and provided with information about the quantitative phase. Participants who agreed to collaborate were formally invited to join the panels. Finally, to standardize the number of members in each panel, 11 individuals were selected from each group.

#### 2-2-2- Knowledge collection from expert panels.

Engaging subject-matter experts in identifying relationships significantly enhances the reliability of the research and leads to more accurate results. To achieve this, a linguistic approach was employed, using subjective linguistic variables. Linguistic variables represent a range of values expressed through natural or artificial language phrases. The linguistic scales and their associated values used in this technique are presented in Table 1. The study utilized triangular fuzzy numbers based on the Chang approach, commonly applied in previous research [22].

**Table 1:** Linguistic expressions and corresponding fuzzy numbers.

Linguistic Expression	Definitive Equivalent	Triangular Fuzzy Numbers
No Impact (No)	1	(0, 0, 0.25)
Very Low Impact (VL)	2	(0, 0.25, 0.5)
Low Impact (L)	3	(0.25, 0.5, 0.75)
High Impact (H)	4	(0.5, 0.75, 1)
Very High Impact (VH)	5	(0.75, 1, 1)

#### 2-2-3- Constructing the pairwise comparison matrix.

**Fig 1:**
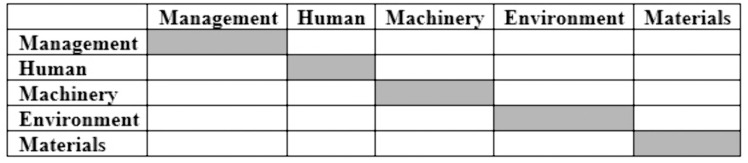
Pairwise comparison matrix for root causes of mining accidents.

Since the identified root causes varied across groups, making complete comparisons between the three groups impractical, the causes were categorized into five groups: management, human, machinery, environment, and materials. A pairwise comparison matrix was created for these five factors (Fig 1).

Pairwise comparison matrices were distributed among relevant individuals, who were asked to use the linguistic variables from Table 1 to express their opinions about the direct influence of each factor on the others.

#### 2-2-4- Data analysis.

After completing the pairwise comparison matrices, the data were extracted and analyzed. The qualitative opinions were converted into fuzzy numbers (Table 1) during the fuzzification process. The analysis proceeded as follows:

1- Constructing the Initial Fuzzy Direct-Relation Matrix ($\tilde{E}$): this matrix was formed by aggregating the opinions of experts. Equation (1), where p represents the number of participating experts, was used to generate the matrix.


$$\tilde{E}=\frac{{\tilde{E}}^{\left\langle \text{1}\right\rangle }+{\tilde{E}}^{\left\langle \text{2}\right\rangle }+\ldots +{\tilde{E}}^{\left\langle p\right\rangle }}{p}\text{ K}=\text{1},\text{2},\ldots \text{p}$$

2- Normalizing the Fuzzy Direct-Relation Matrix: the initial matrix was normalized into a standard matrix using the following equations:


$$\tilde{\text{F}}=\frac{\tilde{\text{E}}}{\gamma }$$

$$\gamma =max\sum_{j=\text{1}}^{n}u_{j}$$

3- Calculating the Fuzzy Total-Relation Matrix: the normalized matrix was expanded to compute the total-relation matrix using algebraic equations (4–6).


$$\text{Matrix}\left[{\text{l}}_{\text{ij}}^{\prime }\right]=\;{\text{F}}_{\text{l}}\times {\left(\text{I}-{\text{F}}_{\text{l}}\right)}^{-\text{1}}$$

$$\text{Matrix}\left[{\text{m}}_{\text{ij}}^{\prime }\right]=\;{\text{F}}_{\text{m}}\times {\left(\text{I}-{\text{F}}_{\text{m}}\right)}^{-\text{1}}$$

$$\text{Matrix}\left[{\text{u}}_{\text{ij}}^{\prime }\right]=\;{\text{F}}_{\text{u}}\times {\left(\text{I}-{\text{F}}_{\text{u}}\right)}^{-\text{1}}$$

4- Defuzzification of the Total-Relation Matrix: Fuzzy data were converted into definitive values using the following equation:


$${\text{t}}_{\text{ij}}=\frac{\text{1}}{\text{4}}\left({\text{l}}_{\text{ij}}^{\prime }+\text{2}{\text{m}}_{\text{ij}}^{\prime }+{\text{u}}_{\text{ij}}^{\prime }\right)$$

5- Calculating Impact (R) and Dependency (D): Defuzzified matrix components were used to calculate the impact and dependency values of each variable using equations (8–9):


$$\text{D}=\sum_{\text{j}=\text{1}}^{\text{n}}{\text{t}}_{\text{ij}}\;,\;(\text{j}=\text{1},\text{2},\text{3}\ldots ,\text{n})$$

$$\text{R}=\sum_{\text{i}=\text{1}}^{\text{n}}{\text{t}}_{\text{ij}}\;,\;(\text{i}=\text{1},\text{2},\text{3}\ldots ,\text{n})$$

6- Establishing Causal Relationships: finally, the indices D + R and D − R were computed. D + R represents the interaction level of a factor with others, while D − R indicates the nature of the interaction. Positive D − R values denote causal factors, while negative values represent dependent factors [12].

## 3- Results

**Fig 2:**
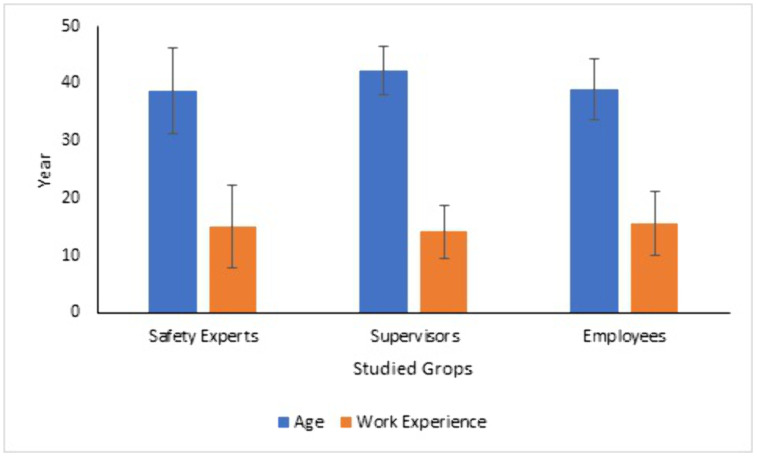
Demographic Characteristics of the Study Population.

In this study, three groups—safety experts, supervisors, and workers—were interviewed. The demographic characteristics of the study population are presented in Fig 2.

### 3-1- Qualitative results

The content analysis of the interviews revealed that the three groups of respondents (workers, supervisors, and safety experts) had differing perceptions regarding the root causes of accidents. The main and sub-themes extracted from the interviews are summarized in Tables 2–4.

**Table 2:** Main and sub-themes extracted from workers’ interviews.

Main Theme	Sub-Themes
Weaknesses in Training Content	1) Mismatch between training courses and job responsibilities.
	2) Lack of easy access to up-to-date and relevant information.
Deficiencies in Safety Equipment Procurement	1) Financial challenges in procuring safety tools.
	2) Delayed or inadequate provision of safety equipment to workers.
Inadequate Workplace Conditions	1) Uneven pathways for machinery.
	2) Poor-quality raw materials.
	3) Generation of dust and smoke.
	4) Workplace noise pollution.
Inappropriate Machinery	1) Lower quality of domestic equipment compared to imported alternatives.
	2) Use of outdated and worn-out equipment.
	3) Failures and issues arising from physical defects.
Human Factors	1) Poor awareness and attitude toward safety.
	2) Lack of periodic performance evaluations.
	3) Absence of proper workplace guidelines.
	4) Insufficient manpower in various mine departments.
Weak Safety Unit Performance	1) Poor introduction of safety unit roles within the organization.
	2) Weak reporting and registration of near misses.
	3) Lack of an incentive and penalty system aligned with safety activities.

**Table 3:** Main and Sub-Themes Extracted from Supervisors’ Interviews.

Main Theme	Sub-Themes
Machinery Deficiencies	1) Limited financial resources for repairs and equipment upgrades.
	2) Lack of regular maintenance for machinery.
	3) Absence of proper instructions for machinery operation.
	4) Economic constraints, sanctions, and political issues affecting the procurement of quality materials and equipment.
Safety Equipment Supply Deficiencies	1) Delayed delivery of safety tools to employees.
	2) Changes in the processes of procurement and delivery of safety equipment.
	3) Non-standard personal protective equipment (PPE).
Harmful Workplace Conditions	1) High levels of dust generation.
	2) Noise caused by machinery.
	3) Adverse regional weather conditions.
	4) Lack of welfare facilities.
Inadequate Training Processes	1) Absence of onboarding training programs.
	2) Lack of appropriate training courses.
	3) Inadequate financial allocation for training.
Psychological and Mental Stress	1) Workforce shortages.
	2) Inadequate employee wages.
	3) Challenges related to adapting to system changes and new processes.
Weak Safety Culture	1) Managerial deficiencies in fostering a safety culture.
	2) Lack of resources, facilities, and facilitation for safety practices.
	3) Unequal treatment of employees regarding safety issues.

**Table 4:** Main and Sub-Themes Extracted from Safety Experts’ Interviews.

Main Theme	Sub-Themes
Managerial System Deficiencies	1) Absence of a scientific and effective management system committed to safety.
	2) Weak managerial mindset regarding the determination of safety objectives and strategies.
Weak Safety Culture Foundations	1) Lack of managerial commitment to fostering a safety culture.
	2) Inadequate understanding among supervisors and managers of the importance of safety.
	3) Poor safety awareness and attitudes among employees.
Inefficient Communication Structures	1) Instability in safety management due to economic sanctions and financial constraints.
	2) Shortages of skilled and committed safety personnel.
	3) Lack of effective communication channels among organizational units.
	4) Absence of comprehensive and unified safety plans to address challenges.
Workforce-Responsibility Mismatch	1) Managers’ lack of awareness of safety challenges.
	2) Employees’ ignorance or neglect of safety issues.
	3) Safety experts’ insufficient knowledge of current safety practices.
Deficiencies in Specialized Training	1) Ineffective onboarding training programs.
	2) Lack of regular and updated training sessions.
Workplace Safety Violations	1) Inadequate use of safety equipment in buildings.
	2) Non-compliance with HSE standards in the workplace.
	3) Adverse regional weather conditions.
Equipment and Machinery Issues	1) Lack of proper maintenance and repair programs.
	2) Insufficient allocation of resources for equipment upgrades.
	3) Financial and resource constraints due to economic sanctions.

Overall, workers emphasized practical and operational challenges such as deficiencies in training content, problems in safety equipment procurement, and adverse workplace conditions. They also drew attention to outdated or inappropriate machinery and human factors, indicating that their perspective was mainly focused on the direct, day-to-day conditions of the work environment.

Supervisors highlighted machinery deficiencies, procurement delays in safety equipment, and inadequate training processes. In addition, they stressed psychological and mental stress factors as well as a weak safety culture. Compared to workers, supervisors provided a broader viewpoint that combined both operational shortcomings and organizational climate issues.

Safety experts focused primarily on systemic and managerial issues, including ineffective management systems, weak safety culture foundations, and inefficient communication structures. They also underscored mismatches between workforce capabilities and job responsibilities, which are less emphasized by workers and supervisors. This perspective reflects their organizational and policy-level role in safety management.

The comparison of root causes from the perspectives of the three groups shows that safety experts emphasized organizational and managerial weaknesses, while supervisors and workers, reflecting their operational roles, primarily identified equipment deficiencies, training inadequacies, and workplace conditions as influential factors. This comparison highlights the differences in viewpoints regarding the root causes of mining accidents.

### 3-2- Quantitative results

In the first phase, the root causes of accidents were identified based on interview analysis, revealing differing perspectives among the three groups. However, in this initial qualitative phase, the relationships between these causes remained unclear. To address this limitation, the fuzzy DEMATEL method was employed to determine the causal relationships among the factors.

After deriving the initial fuzzy direct-relation matrix, the normalized fuzzy direct-relation matrix and the fuzzy total-relation matrix were computed for the three groups. In the next step, the fuzzy total-relation matrix was defuzzified to obtain comparable values (Tables 5–7).

**Table 5:** Defuzzified Total-Relation Matrix for Workers.

TD	A	B	C	D	E
A	0.03	0.13	0.11	0.11	0.10
B	0.10	0.04	0.11	0.12	0.10
C	0.07	0.11	0.03	0.11	0.10
D	0.09	0.12	0.10	0.03	0.10
E	0.07	0.12	0.10	0.11	0.03

**Table 6:** Defuzzified Total-Relation Matrix for Supervisors.

TD	A	B	C	D	E
A	0.03	0.13	0.12	0.11	0.10
B	0.11	0.04	0.12	0.11	0.10
C	0.10	0.12	0.04	0.11	0.10
D	0.10	0.13	0.10	0.03	0.09
E	0.09	0.12	0.11	0.10	0.03

**Table 7:** Defuzzified Total-Relation Matrix for Safety Experts.

TD	A	B	C	D	E
A	0.85	1.10	1.02	1.07	1.01
B	0.91	0.81	0.89	0.95	0.87
C	0.85	0.91	0.70	0.87	0.82
D	0.87	0.94	0.83	0.75	0.83
E	0.85	0.92	0.86	0.91	0.71

The average scores of the defuzzified total-relation matrix were considered as the threshold. The thresholds were 0.094 for workers, 0.098 for supervisors, and 0.888 for safety experts. Accordingly, an influence score of ≥0.094 for workers, ≥ 0.098 for supervisors, and ≥0.888 for safety experts indicated a significant causal influence of one factor on another.

In the subsequent stage, the values for D (influence), R (dependence), D + R, and D-R were calculated to assess the influence and dependency of factors (Tables 8–10). D + R indicates the level of interaction a factor has with other factors. D-R determines whether a factor acts as a cause (positive D-R) or an effect (negative D-R).

**Table 8:** Fuzzy DEMATEL Values for Workers.

Variable	D	R	D + R	D-R	Role
Management	0.50	0.38	0.88	0.12	Cause
Human	0.50	0.53	1.03	−0.03	Effect
Machinery	0.44	0.48	0.92	−0.04	Effect
Environment	0.46	0.51	0.97	−0.05	Effect
Materials	0.45	0.46	0.91	−0.01	Effect

**Table 9:** Fuzzy DEMATEL Values for Supervisors.

Variable	D	R	D + R	D-R	Role
Management	0.51	0.46	0.97	0.05	Cause
Human	0.50	0.56	1.06	−0.06	Effect
Machinery	0.50	0.51	1.01	−0.01	Effect
Environment	0.47	0.49	0.96	−0.02	Effect
Materials	0.47	0.45	0.92	0.02	Cause

**Table 10:** Fuzzy DEMATEL Values for Safety Experts.

Variable	D	R	D + R	D-R	Role
Management	5.07	4.35	9.42	0.72	Cause
Human	4.45	4.71	9.16	−0.26	Effect
Machinery	4.18	4.32	8.50	−0.14	Effect
Environment	4.24	4.58	8.82	−0.34	Effect
Materials	4.28	4.26	8.54	0.02	Cause

The fuzzy DEMATEL results indicate similar perceptions among the three groups regarding the causal relationships between root causes of accidents. All groups identified management as a cause, while human factors, machinery, and environment were recognized as effects. However, there was a discrepancy regarding materials: workers viewed it as an effect, whereas safety experts and supervisors considered it a cause.

## 4- Discussion

The qualitative phase of the study revealed that workers, supervisors, and safety experts had differing perspectives on the root causes of mining accidents. However, all three groups commonly emphasized deficiencies in training, machinery and safety equipment, and the workplace environment as critical factors.

Safety training is one of the most fundamental tools for preventing mining accidents. However, deficiencies in delivering adequate and effective training can lead to risky behaviors, reduced awareness, and increased accident rates. Inadequate training leaves workers unaware of the hazards present in the mining environment, potentially leading to risky behaviors and neglect of safety measures. For instance, a lack of familiarity with safe work practices in confined spaces or areas prone to collapses significantly increases the risk of severe incidents [23].

One of the primary goals of safety training is to equip workers with the necessary skills to identify and respond appropriately to hazardous conditions. In the absence of effective hands-on training, workers may fail to make timely and appropriate decisions during emergencies, such as explosions or collapses. This deficiency can exacerbate the severity of accidents [24].

Deficiencies in training programs also contribute to the development or reinforcement of a weak safety culture within mining environments. In workplaces where adequate training is not provided, both workers and managers often display a diminished attitude toward the importance of safety. This lack of emphasis on safety leads to non-compliance with safety regulations and negligence of procedures [25].

Aged machinery, due to prolonged use and frequent operation, often experiences reduced reliability and an increased likelihood of failure. These failures can lead to catastrophic incidents in critical environments, such as underground mines with limited ventilation. Studies indicate that over a quarter of mining accidents are caused by equipment malfunctions, often stemming from machinery wear and tear [26]. Mining equipment, including drilling, transportation, and ventilation systems, are essential components of the extraction process. Failures in these systems can disrupt mining operations and heighten sudden risks, such as tunnel collapses, vehicle accidents, or gas explosions. Research has shown that equipment malfunctions account for more than 25% of mining accidents [26].

Access to standard safety equipment is a key factor in ensuring worker safety, and its absence can directly jeopardize the health and safety of personnel [27]. Safety equipment, such as gas sensors, respiratory masks, and fire suppression systems, must function effectively to prevent serious hazards. For instance, inaccurate sensors may fail to detect hazardous gases like methane or carbon monoxide in time, potentially leading to explosions or asphyxiation [28].

Another common cause is insufficient attention to workplace conditions. Due to their unique nature, working environments in mines are critical factors in the occurrence of accidents. Factors such as geographical conditions, inadequate ventilation, poor lighting, and the presence of hazardous materials can endanger worker safety and increase the likelihood of accidents. Reports indicate that over 20% of mining accidents result from the accumulation of hazardous gases, directly linked to weaknesses in ventilation systems [28].

Mining environments, particularly in mountainous or deep underground areas, present specific geographical risks. Unstable ground conditions can lead to tunnel collapses, while unmanaged groundwater can heighten the risk of flooding or landslides [29]. Insufficient lighting in underground workplaces increases the likelihood of accidents, such as collisions with machinery, falls, or other incidents. Studies have shown that proper lighting can reduce accident rates by up to 30%, while inadequate lighting is a primary factor in many risky behaviors [30].

The presence of hazardous minerals or chemicals in mines, such as silica or toxic gases, poses significant health risks to workers. Prolonged exposure to these substances not only leads to chronic risks, such as lung diseases, but under certain conditions, can also cause sudden incidents, such as explosions or toxic material leaks [31].

In addition to the common causes among the three groups, employees identified human resources and the poor performance of the safety unit as root causes of mining accidents. A workforce with insufficient awareness and a negative attitude toward safety, coupled with a lack of adequate personnel in various sections of the mine, were highlighted by staff as significant contributing factors. Workers who are unaware of the consequences of hazards or fail to understand the importance of adhering to safety guidelines are more prone to engaging in risky behaviors. Studies indicate that low levels of awareness and a negative attitude toward safety can increase the likelihood of accidents by up to 30% [32].

Finally, the poor performance of the safety unit was another issue highlighted by employees. Problems such as inadequate communication of the unit’s activities and responsibilities, weaknesses in reporting issues, and the absence of an effective reward and penalty system aligned with safety activities were noted. Establishing a robust and transparent management system within the safety unit can help improve safety culture and reduce accidents [33].

Effective oversight and enforcement of safety regulations are key responsibilities of the HSE unit. When such oversight is poorly executed or periodic inspections are delayed, minor hazards can escalate into major incidents. For example, failure to address technical issues in machinery or to monitor the proper use of protective equipment significantly increases the risk of accidents [34].

The results of interviews with supervisors revealed that this group emphasized deficiencies in safety culture and psychological stress. The shared focus of both supervisors and safety officers on the deficiency in safety culture highlights its fundamental role in the occurrence of accidents. Safety culture is a key element in ensuring the health and safety of workers and reducing accidents in high-risk environments like mines. A deficiency in safety culture, characterized by a lack of organizational and individual commitment to safety principles, can exacerbate workplace hazards and lead to serious incidents.

Deficient safety culture often correlates with the low prioritization of safety at both managerial and operational levels. This issue can result in reduced investment in safety equipment, inadequate training, and weak oversight of compliance with safety protocols. Research shows that organizations with weak safety cultures have higher accident rates [35]. In environments lacking a strong safety culture, workers’ attitudes and behaviors toward safety may be indifferent or negative. These attitudes can lead to risky behaviors, such as ignoring safety protocols or failing to use protective equipment. Studies indicate that such risky behaviors are a major factor in mining accidents [36].

One characteristic of a weak safety culture is the fear of reporting accidents or near-misses, stemming from concerns about punishment or the dismissal of reports. This deficiency can result in missed opportunities to learn from past incidents and prevent similar accidents in the future [37]. A weak safety culture is typically associated with systemic shortcomings at various organizational levels. These include the absence of clear safety policies, insufficient resources for monitoring operations, and a lack of ongoing worker training. Collectively, these factors significantly increase the likelihood of accidents [38].

Psychological and mental strains are among the key factors contributing to reduced worker safety and increased accident rates in mines. These stresses often stem from issues such as labor shortages, inadequate wages, and constant changes in systems and work processes.

Labor shortages in mines place additional workloads on the remaining workers. This added pressure can lead to both physical and mental fatigue, negatively affecting workers’ decision-making and accuracy. Studies have shown that occupational fatigue is a contributing factor in over 20% of mining accidents [28].

Insufficient wages can result in decreased motivation and dissatisfaction among workers. These negative emotions may lead to reduced adherence to safety protocols and an increase in risky behaviors. Research indicates that workers with low job satisfaction are more likely to make errors, which can result in unfortunate accidents [38]. Frequent changes in technology, work systems, or operational procedures can create psychological stress, making it challenging for workers to adapt. These conditions reduce focus and increase the likelihood of human error. The risk of accidents rises significantly, especially when adequate training to adapt to these changes is not provided [23]. The combined effects of labor shortages, inadequate wages, and constant changes often exacerbate negative outcomes. These factors contribute to reduced mental health, increased stress, and non-standard behaviors, all of which are significant contributors to mining accidents [24].

From the perspective of safety managers, in addition to the shared causes of mining accidents, deficiencies in management systems, ineffective communication structures, and mismatches between workforce capabilities and job responsibilities play significant roles in accident occurrence. An effective and scientific management system in mines is crucial for reducing accidents and improving workplace safety.

The absence of a scientific and efficient management system committed to safety, coupled with shortcomings in managerial thinking when setting safety goals and strategies, can heighten the risk of accidents. Scientific safety management requires structured processes and data-driven methods to identify and mitigate hazards. Without such a system, decisions are often made without considering safety principles, leaving potential risks unaddressed. Research indicates that organizations with inefficient safety management systems experience higher accident rates [39]. Poor managerial thinking can lead to setting inappropriate goals and failing to formulate effective strategies for safety. Without clear objectives and strategies, operational units cannot work cohesively to mitigate risks. This lack of alignment results in gaps in implementing safety protocols, increasing the likelihood of accidents [38]. Managerial commitment to safety is a key factor in creating a safe working environment. When senior management does not prioritize safety issues adequately, this attitude permeates lower levels of the organization, weakening the safety culture. A weak safety culture fosters risky behaviors and neglect of safety principles among workers [37].

Effective communication plays a vital role in identifying hazards, coordinating operations, and reducing the occurrence of accidents in mining environments. Inefficient communication structures can lead to the miscommunication of information, a lack of coordination between units, and delayed responses to hazardous situations. In mining operations, the rapid and accurate exchange of information about risks—such as collapses, the buildup of dangerous gases, or equipment malfunctions—is of critical importance. In inefficient communication systems, information is either not conveyed correctly or is delayed, which can result in severe accidents. Studies have identified communication failures as one of the primary contributors to mining incidents [40]. The absence of effective communication channels between various mining units, such as extraction teams, ventilation units, and supervisory teams, can disrupt operational coordination. This lack of coordination is particularly dangerous during emergencies, such as fires or tunnel collapses, where it can amplify the severity and scope of the incident [36].

The alignment between the abilities of the workforce and job responsibilities plays a crucial role in reducing accidents and improving productivity in mining operations. A mismatch in skills, experience, or physical and psychological capacities with job requirements can lead to operational errors and serious incidents. Certain mining tasks demand specific physical or psychological capabilities. Workers who are not adequately equipped for these tasks may exhibit poor performance under pressure or fatigue. This can result in risky behaviors or an inability to respond quickly to hazardous situations [28]. At the managerial level, assigning individuals without sufficient knowledge of safety or mining operations can lead to poor decision-making and increased risks. Similarly, at the operational level, allocating tasks without considering the experience or skills of workers raises the likelihood of human error and accidents [40]. A mismatch between workforce capabilities and job demands can increase stress, job dissatisfaction, and psychological fatigue. These conditions directly affect focus and adherence to safe practices, escalating the risk of accidents. Research indicates that occupational stress is a significant factor in mining incidents [38].

Effective accident management and prevention in organizations require coordination among individuals at various organizational levels. Differences in perspectives between managers, supervisors, and employees can lead to varied understandings of the causes, consequences, and solutions to incidents. If not properly managed, these differences can undermine the effectiveness of prevention and control strategies and increase the risk of accidents. When perspectives are not aligned, root cause analyses of accidents may remain incomplete. For instance, managers might focus on systemic deficiencies, while supervisors prioritize human behavior. This lack of cohesion in focus can result in incomplete solutions that fail to address all contributing factors [40].

Overall, differences in perspectives among experts, supervisors, and operators regarding the root causes of accidents can lead to more comprehensive problem identification and the development of creative and effective solutions. However, mismanagement of these differences may result in misalignment and reduced organizational acceptance. By improving internal communication, creating opportunities for active participation, and applying relevant scientific theories, organizations can leverage these differences as a valuable resource to enhance safety.

A study using the fuzzy DEMATEL method, conducted with input from three groups—workers, supervisors, and safety experts—on the root causes of mining accidents revealed significant similarities in the cause-and-effect interactions and interdependencies between various factors. The findings showed that all three groups recognized management as the primary and most influential factor in the occurrence or prevention of accidents, while other factors such as human behavior, environment, and machinery were identified as secondary or dependent factors. This perspective is rooted in the scientific understanding of management’s pivotal role in safety systems and its influence on other factors.

Management serves as the cornerstone of any organizational system, responsible for formulating, implementing, and overseeing safety policies, procedures, and programs. Managerial decisions directly impact resource allocation, employee training, equipment maintenance planning, and the assurance of workplace quality. This influence shows that deficiencies in management can affect all other factors, ultimately increasing the likelihood of accidents [39].

One critical aspect of management’s impact is its role in shaping the organization’s safety culture. Safety culture encompasses the values, attitudes, and behaviors that prioritize safety. Through policymaking, support, and leading by example, management can either strengthen or weaken this culture. Research by Hollnagel (2014) indicates that organizations with stronger safety cultures not only experience fewer accidents but also exhibit better responses to emergencies and unexpected hazards [7].

The human factor, identified as one of the primary dependent variables, is directly influenced by management decisions. By providing adequate training, designing a safe working environment, and ensuring the availability of safety equipment, management can significantly reduce the likelihood of human error [6,41,42].

The work environment, machinery, and materials are also identified as factors influenced by management. Poor equipment maintenance, the use of low-quality or hazardous materials, and inadequate management of environmental conditions often result from insufficient resources or misaligned managerial priorities. The interactions among system components (e.g., management, human factors, and environment) are largely regulated by managerial decisions. Any deficiencies in these interactions can lead to accidents [2].

These findings align with systems theory, which views management as the primary controller responsible for regulating relationships between system components. Any shortcomings in this control can lead to system disarray and heightened risks. This approach underscores why management is seen as the root cause, with other factors acting as dependent variables [2].

One notable distinction in the analysis of causal relationships pertains to materials: safety experts and supervisors identified materials as a root cause, while workers regarded them as a dependent factor. This difference arises from the varying roles and experiences of these groups.

Despite differences in views regarding certain root causes, the study revealed a relatively common understanding of the relationships between causes. This shared perspective is crucial for aligning managerial approaches and improving safety strategies. Harmonized views across groups can significantly enhance the effectiveness of safety programs [43].

The variations in perspectives highlight the need for management strategies that consider the unique roles, needs, and priorities of each group. Understanding and analyzing these viewpoints is essential for creating safety strategies that not only address core safety issues but also align with the diverse expectations of all stakeholders. By fostering mutual understanding and coordination between groups, organizations can develop more comprehensive and effective safety programs that reduce accidents and improve workplace safety.

The findings of this study have important managerial implications. Since management was consistently identified as the most influential factor, managers can play a pivotal role in accident prevention by strengthening organizational safety culture, allocating sufficient resources for equipment maintenance, and ensuring the provision of standard safety equipment. They should also design and support targeted training programs that address both technical and behavioral aspects of safety. In addition, by fostering open communication channels and actively engaging workers and supervisors in safety decision-making, managers can bridge the gap between different perspectives and build a shared understanding of risks. Implementing these measures can not only reduce accident rates but also improve productivity, job satisfaction, and trust in the organization’s safety system.

Despite its strengths, this study has several limitations. First, the qualitative phase relied on self-reported interview data, which may be subject to recall bias or social desirability bias. Although measures such as member checking and expert review were applied to enhance rigor, these biases cannot be entirely excluded. Second, the study was conducted in a specific mining context, which may limit the generalizability of findings to other mining industries or geographic regions. Future research could address these limitations by incorporating observational data and replicating the study across different mining settings.

## 5- Conclusion

Identifying the root causes of mining accidents and analyzing the causal relationships between them from the perspectives of different workgroups plays a crucial role in improving safety management systems. The findings of this study revealed that the diverse perspectives of workers, supervisors, and safety officials each highlight key contributing factors to accidents, and together provide a more comprehensive view of the root causes.

On the one hand, workers, due to their direct experience with the work environment and equipment, identified operational and environmental deficiencies. Supervisors, meanwhile, emphasized factors related to human behavior and the lack of adequate equipment. Safety officials, adopting a more systematic approach, focused on managerial and organizational weaknesses. These differences highlight the importance of integrating multiple perspectives to achieve a more comprehensive accident analysis.

The causal analysis using the fuzzy DEMATEL method identified management as the primary factor influencing the occurrence or prevention of accidents. In contrast, factors such as human behavior, environment, and machinery were more often viewed as dependent variables. These findings highlight the importance of focusing on management as the starting point for safety reforms, given its direct impact on other factors. This study emphasizes that integrating the perspectives of different groups and analyzing causal relationships between root causes can support the development of more comprehensive and effective preventive strategies. Such an approach not only reduces accidents and improves safety but also enhances worker productivity and job satisfaction.
