# Correction: Root cause analysis of accidents and examining their interrelations from the perspective of workers, supervisors, and safety officers

**DOI:** 10.1371/journal.pone.0357924

**Published:** 2026-09-08

**Authors:** Neda Molamehdizadeh, Gholam Hossein Halvani, Hossein Ebrahimi, Ali Asghar Farshad, Seyedeh Melika Kharghani Moghadam, Tahereh Eskandari

There is an error in references 26, 27, 29, 30, 31, 33, and 43. The correct references are:

26. Ruff T, Coleman P, Martini L. Machine-related injuries in the US mining industry and priorities for safety research. Int J Inj Contr Saf Promot. 2011;18(1):11-20.
27. Bansah KJ, Yalley AB, Dumakor-Dupey N. The hazardous nature of small scale underground mining in Ghana. J Sustain Min. 2016;15(1):8-25.
29. Satyanarayana I, Sinha AK. A critical review of stability analysis and design of pit slopes in Indian opencast coal mines. Chem Eng Trans. 2018;66:1231-1236.
30. Elvik R. Meta-analysis of evaluations of public lighting as accident countermeasure. Transp Res Rec. 1995;1485(1)12-24.
31. Hahn M. Safety and health in underground coalmines: ILO code of practice.
33. Robson LS, Clarke JA, Cullen K, Bielecky A, Severin C, Bigelow PL, et al. The effectiveness of occupational health and safety management system interventions: A systematic review. Saf Sci. 2007;45(3):329-353.
43. Hale AR, Guldenmund FW, van Loenhout PLCH, Oh JIH. Evaluating safety management and culture interventions to improve safety: Effective intervention strategies. Saf Sci. 2010;48(8):1026-1035.
